# Hyperthyroidism Is Associated with the Development of Vasospastic Angina, but Not with Cardiovascular Outcomes

**DOI:** 10.3390/jcm9093020

**Published:** 2020-09-19

**Authors:** Hyun-Jin Kim, Sang-Ho Jo, Min-Ho Lee, Won-Woo Seo, Sang Hong Baek

**Affiliations:** 1 Division of Cardiology, Department of Internal Medicine, Hanyang University College of Medicine, Seoul 04763, Korea; titi8th@hanyang.ac.kr; 2 Division of Cardiology, Department of Internal Medicine, Hallym University Sacred Heart Hospital, Anyang-si 14068, Korea; 3 Division of Cardiology, Department of Internal Medicine, Soonchunhyang University Seoul Hospital, Seoul 04401, Korea; neoich@gmail.com; 4 Division of Cardiology, Department of Internal Medicine, Kangdong Sacred Heart Hospital, Hallym University College of Medicine, Seoul 05355, Korea; wonwooda@gmail.com; 5 Division of Cardiology, Seoul St. Mary’s Hospital, The Catholic University of Korea, Seoul 06591, Korea; whitesh@catholic.ac.kr

**Keywords:** vasospastic angina, hyperthyroidism, clinical outcomes

## Abstract

Vasospastic angina (VA) is a functional disease caused by the alteration of vasomotor tone. We investigated the association of hyperthyroidism with the development and prognosis of VA. Study data were obtained from a prospective multicenter registry that included patients who had symptoms suggestive of VA. Coronary angiography and an ergonovine provocation test were performed, and patients were classified into a VA and a non-VA group. Among 1239 patients with suspected VA, 831 patients were classified in the VA group. Hyperthyroidism was more common in the VA group than in the non-VA group (10.0% vs. 3.7%, *p* < 0.001). After adjusting for confounding factors, hyperthyroidism was independently associated with a 3.27-fold increased risk of VA. Especially in women, hyperthyroidism was associated with a 4.38-fold higher risk of VA. All-cause death rates did not differ according to the presence or absence of hyperthyroidism. Hyperthyroidism is independently associated with the occurrence of VA especially in women but did not affect the total death in VA patients. Clinicians need to be aware of the role of thyroid function in patients with suspected VA.

## 1. Introduction

Vasospastic angina (VA) is a functional disease caused by the focal or diffuse spasm of the smooth muscle layer of the coronary arterial wall, causing a high grade of obstruction. VA has survival rates of 98 and 93% at 1 and 10 years, and a myocardial infarction-free survival of 86 and 81% at 1 and 10 year follow-up [1,2]. The prevalence of VA has not been extensively studied, but it appears to be more common in Korean and Japanese populations than in Caucasian ones [3,4].

Several studies of patients with VA have shown that high blood pressure and dyslipidemia, which are the traditional risk factors of atherosclerotic cardiovascular disease, are not significantly associated with vasospasm, but smoking is the only proven risk of vasospasm [5,6]. A recent study demonstrated that hyperthyroidism is associated with increased risk of developing myocardial infarction and stroke independent of atherosclerotic risk factors, but is not associated with mortality in a large scale cohort [7] Hyperthyroidism has been known to increase vasomotor activity and up-regulate numbers of adrenergic receptors, resulting in enhanced sympathoadrenal activity [8,9]. When we consider that the VA is a disease caused by the alteration and imbalance of vasomotor tone [10], we can assume that the thyroid hormone, which influences the sympathetic tone and vascular constriction/relaxation, is more likely to be related to the development and the prognosis of VA than to atherosclerotic ischemic heart disease. However, there has been no study pursuing the relationship of thyroid hormone and vasospasm, except for a few case reports, which demonstrated that an excess of thyroid hormone caused severe forms of spasm invading the left main coronary artery, particularly in women [3,11,12,13]. We investigated for the first time, as far as we know, the association between hyperthyroidism and VA development and its impact on the prognosis of VA using a prospective, multi-center, large scale cohort from Korea.

## 2. Materials and Methods

### 2.1. Study Population

Study data were obtained from the Vasospastic Angina in Korea registry (VA-Korea), which is a prospective multi-center registry. The study design and the primary results have been published previously [2,10,14,15]. In brief, eleven tertiary hospitals in Korea participated in this registry between May 2010 and June 2015; patients aged >18 years with symptoms of suspected VA were tested by invasive coronary angiography (CAG) and an ergonovine (EG) provocation test and enrolled in the study. Patients with normal or mild coronary atherosclerosis (<50% luminal diameter narrowing according to the baseline CAG) could be enrolled, but patients with significant coronary atherosclerosis (≥50% luminal diameter narrowing) were excluded from the study. Patients with known malignancy or inflammatory disease, end-stage renal disease on continuous dialysis, or catheter-induced spasm at baseline were excluded. Among the initially enrolled 2960 patients with suspected VA without fixed lesion, 1239 patients had available data on thyroid function from blood tests and were included in the final analysis. This study protocol complied with the Declaration of Helsinki and was reviewed and approved by the Institutional Review Board of Hallym University Sacred Heart Hospital (Approved No. 2010-I007, 25 Mar 2010). All patients gave written informed consent prior to study entry.

### 2.2. Data Collection

The patient data were collected from the VA-Korea database via a web-based electronic data capture system that included an electronic case report form. The following patient demographic and clinical characteristics were extracted from this database: age, body mass index (BMI; kg/m^2^), blood pressure, traditional cardiovascular risk factors including alcohol drinking and current smoking status, and previous cardiovascular medications. Each patient’s BMI was calculated from his or her height and weight. Major laboratory data were also collected at admission: thyroid-stimulating hormone (TSH), T3 and free T4. We also extracted left ventricular ejection fraction from echocardiography data to determine left ventricular systolic function at admission.

The primary outcome was the development of VA. The secondary outcomes comprised cardiac death, acute coronary syndrome, and new-onset arrhythmia including ventricular tachycardia, ventricular fibrillation, and atrioventricular (AV) block, and all cause death during follow-up (median duration, 832 days; mean duration, 763 days). The occurrence of death and the timing of death were confirmed through a review of medical records or from a telephone interview.

### 2.3. Measurement of Thyroid Hormone and Definition of Hyperthyroidism

The serum level of TSH, free T4, was measured routinely in each hospital with the standard method and the T3 level was at the physician’s discretion. Hyperthyroidism was defined as a suppressed TSH level and an increased serum free T4 and/or T3 concentration based on local, assay-specific reference ranges according to the 2016 American Thyroid Association Guidelines [16,17], and determined by the judgement of the clinicians at each center. In patients without a T3 level, hyperthyroidism was defined as a suppressed TSH level and elevated levels of free T4. The elevation of serum TSH, free T4 and T3 was judged by doctors following each hospital’s cut-off value.

### 2.4. Invasive CAG and EG Provocation Test

The baseline CAG and EG provocation tests were performed according to the Guidelines for Diagnosis and Treatment of Patients with VA of the Japanese Circulation Society [18]. Detailed methods of CAG, the EG provocation test and adjudication of EG provocation test results were reported previously [2,10].

Significant vasospasm was defined as total or luminal diameter narrowing over 90% of the coronary arteries accompanied by chest pain and/or ECG change after intracoronary EG injection [18,19]. Intermediate spasm was defined as a 50% to 90% luminal diameter narrowing of coronary arteries. Non-VA was defined as less than 50% luminal narrowing without chest pain or ECG change. On the basis of these findings, patients were classified into two groups: the VA group (including patients with significant vasospasm and intermediate spasm) and the non-VA group. Then, these patients were sub-divided into two groups, again according to the presence or absence of hyperthyroidism. All patients who had spasms on the EG provocation test or spontaneous spasm were treated with medication, including calcium-channel blockers and other vasodilators, during follow-up according to the clinician’s discretion.

### 2.5. Statistical Analyses

All categorical data are presented in frequencies and percentages, continuous variables with normal distribution are expressed as means and standard deviations, and continuous variables with non-normal distribution are presented as median (interquartile range). For continuous variables, the Shapiro–Wilk test was used for confirming the normal distribution of each dataset. Pearson’s chi-squared test was used to compare categorical variables, the Student’s *t*-test was used to compare continuous variables with normal distribution, and the Mann–Whitney U test was used to compare continuous variables with non-normal distribution. Kaplan–Meier survival analyses and log-rank tests were used to compare cumulative composite clinical event-free survival rates according to whether the patient had hyperthyroidism or not.

We divided the patients into 4 groups according to the highest quartile/lowest quartile of TSH and VA status and performed survival analysis to see the dose-dependent manner of thyroid function on clinical outcome. Same analysis was performed with free T4 and T3.

In addition, univariate analysis and subsequent multivariable logistic regression analysis were performed to assess the risk of VA after adjustment for individual risk factors. For evaluating the difference in the effect of hyperthyroidism on VA according to sex, a *p*-for interaction between sex and hyperthyroidism was provided using logistic regression analysis. Variables with predictive significance (*p* < 0.05) of VA in univariate analysis were included in the regression analysis.

In addition, TSH, free T4, and T3 were treated as continuous variables in the model, and restricted cubic spline function was used to investigate the non-linearity relationship between predictors and outcomes, and showed the plots for demonstrating the relationship. A *p*-value < 0.05 was considered statistically significant. All analyses were performed using SPSS 21.0 software (IBM Corp., Armonk, NY, USA) and also using the statistical software R-3.5.2 (Vienna, Austria).

## 3. Results

### 3.1. Baseline Characteristics

Among 1239 patients (629 men and 610 women) with suspected VA who underwent CAG and EG provocation tests, 831 (67.1%) had VA and the others did not have VA. Among 629 men and 610 women, 485 (77.1%) and 346 (56.7%) had VA, respectively. Patients’ baseline characteristics according to VA status are shown in Table 1. Alcohol drinking and current smoking were significantly more common in the VA group than in the non-VA group.

TSH was significantly lower (*p* = 0.013) and free T4 was significantly higher (*p* < 0.001) in the VA group. Serum T3 level was available only in 820 patients (66.1%) and was similar between the two groups (2.0 vs. 2.3 ng/dL, VA vs. non-VA, *p* = 0.631). The rate of hyperthyroidism was also significantly higher in patients with VA as compared to those with non-VA (10.0% vs. 3.7%, *p* < 0.001). Supplementary Table S1 shows the baseline characteristics of patients according to sex. In both sexes, current smoking was more frequent in the VA-group than the non-VA group.

### 3.2. Effect of Hyperthyroidism on VA

**Figure 1:**
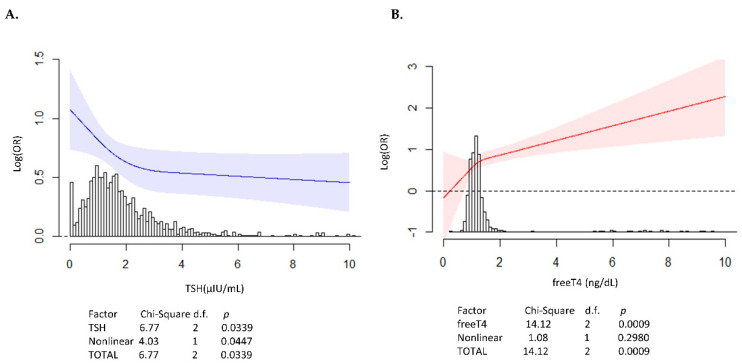
The association for TSH, free T4 level with VA development. (**A**) Plots of the estimated restricted cubic spline function relating TSH to VA development. The log-transformed odds of VA increased as the TSH level decreased, and the TSH level of 2 μIU/mL was the knot and inflection point. There was a non-linear association between log-transformed odds of VA and TSH (*p*-value = 0.0447). The solid line represents the estimated log OR of VA development, and the shaded area is 95% CI. Below is a density plot showing the distribution of observed TSH. (**B**) Plots of the estimated restricted cubic spline function relating free T4 to the VA. The log-transformed odds of VA increased as the free T4 level increased with linear association. The solid line represents the estimated log OR of VA development, and the shaded area is 95% CI. Below is a density plot showing the distribution of observed free T4. The dotted line represents when the OR of VA development is 1. CI, confidence interval; OR, odds ratio; TSH, thyroid-stimulating hormone; VA, vasospastic angina;T3, triiodothyronine;T4, thyroxine.

According to univariate analysis, the following factors were linked with VA: hyperthyroidism (odds ratio (OR): 2.91, 95% confidence interval (CI): 1.655–5.106, *p* < 0.001), serum free T4 level, male sex, current smoking and alcohol drinking (Table 2). TSH level showed a significant non-linear association with the log-transformed odds of VA development using restricted cubic spline with three knots (*p* = 0.045) (Figure 1A), and free T4 level showed a linear association with VA (Figure 1B). However, T3 level was not associated with VA development, whether linear or non-linear. After adjusting for confounding factors, the multivariable logistic regression analysis showed that hyperthyroidism was independently associated with a 3.27-fold increased risk of VA (OR: 3.27, 95% CI: 1.811–5.897, *p* < 0.001) (Table 2). Male sex and current smoking were also significantly related with VA.

There was no significant difference in the effect of hyperthyroidism on VA between men and women (*p* or interaction between sex and hyperthyroidism was 0.314). Table S2 shows the predictors of VA according to sex. Hyperthyroidism was independently associated with a 4.38-fold increased risk of VA in women (OR: 4.38, 95% CI: 2.011–9.537; *p* < 0.001) (Table S2).

### 3.3. Clinical Outcomes in Patients with VA

**Figure 2:**
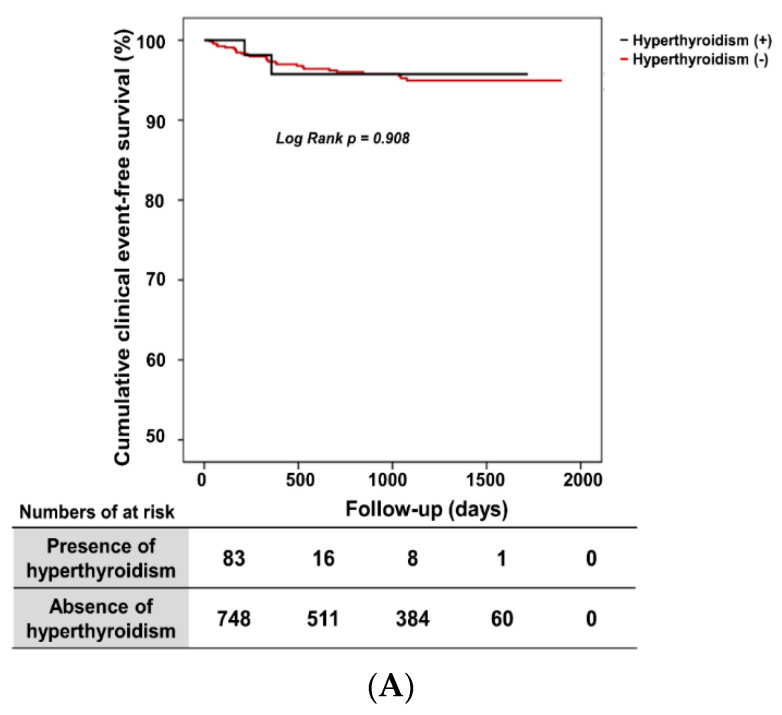

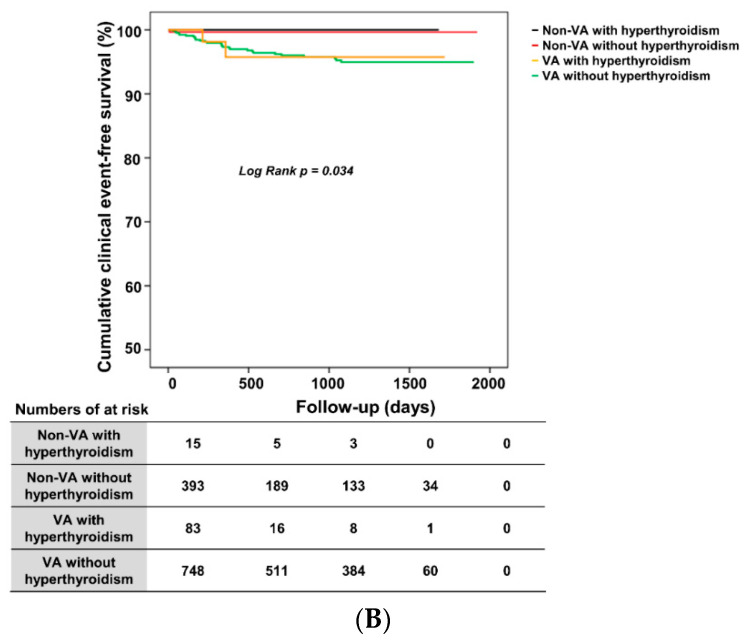
Cumulative composite clinical event-free survival rates according hyperthyroidism status. (**A**) There was no significant difference in cumulative composite clinical event-free survival rate between the VA with hyperthyroidism group and the VA without hyperthyroidism group after long-term follow-up. (**B**) The VA group with hyperthyroidism and the VA group without hyperthyroidism had significantly worse event-free survival after long-term follow-up. VA, vasospastic angina.

Cumulative composite event-free survival rates between the presence- and absence-of-hyperthyroidism groups did not differ in VA patients (97.5% vs. 96.2%, long-rank *p* = 0.908, Figure 2A).

Thirty patients (3.6%) in the VA group experienced clinical events during the follow-up (Table 3). The composite clinical events rate of cardiac death, acute coronary syndrome, new-onset arrhythmia, or AV block was not different between the presence- and absence-of-hyperthyroidism groups in VA patients, 2.4% vs. 3.7%, respectively (*p* = 0.537). A total of 4 patients in the VA group died during follow-up (0.0% vs. 0.5% in presence- vs. absence-of-hyperthyroidism, *p* > 0.999).

In both men with VA and women with VA, the composite clinical event rates did not differ according to the presence or absence of hyperthyroidism (Men; 2.4% vs. 3.6%, *p* > 0.999, women; 2.4% vs. 3.9%, *p* > 0.999) (Table S3). In addition, there was no difference in the effect of hyperthyroidism on composite clinical outcomes between men and women (*p* for interaction between sex and hyperthyroidism was 0.947).

Figure 2B shows the cumulative composite clinical event-free survival rates according to hyperthyroidism status in both the VA group and the non-VA group. The VA group with hyperthyroidism and the VA group without hyperthyroidism had significantly lower cumulative composite event-free survival rates (long-rank *p* = 0.034), i.e., the VA group, irrespective of hyperthyroidism status, had worse clinical outcomes as compared to the non-VA group. The presence of hyperthyroidism in both the VA and non-VA group did not affect the clinical outcome (Figure 2B), i.e., the clinical outcome between the VA with hyperthyroidism and the VA without hyperthyroidism group did not differ. Moreover, the clinical event rate was not different between the non-VA with hyperthyroidism and the non-VA without hyperthyroidism group.

When we performed survival analysis according to the four groups classified by the thyroid function (quartile of each TSH, free T4 and T3) and VA status (i.e., VA—highest quartile of thyroid function, VA—lowest quartile of thyroid function, non-VA—highest quartile of thyroid function and non-VA—lowest quartile of thyroid function), VA with the highest quartile of TSH patients showed a trend for lower event-free survival as compared to those with VA with the lowest quartile of TSH and non-VA patients irrespective of TSH quartile (Figure S1A). In the view point of T3, the same trend for worse outcomes was detected in patients with VA with the highest quartile of T3 (Figure S1B). These differences were not statistically significant. There was no difference among the four groups in terms of free T4 classification. (Figure S1C).

## 4. Discussion

According to results from this prospective multi-center large-scale registry, the incidence of hyperthyroidism was significantly higher in patients with VA than in patients without VA. Hyperthyroidism was also independently associated with a significantly increased risk of VA occurrence (as much as 3.27-fold). The prognosis of VA was excellent in both men and women, and there was no difference in clinical outcomes according to the presence of hyperthyroidism.

Thyroid function is related with cardiovascular hemodynamics. First, thyroid hormone increases consumption of peripheral oxygen and substrate requirements, leading to an increased cardiac contractility, as well as directly increasing cardiac contractility [20,21]. Second, the direct effect of thyroid hormone on vascular smooth muscle cells causes vasodilation and reduces vascular resistance [22]. As a result of a decreased systemic vascular resistance, the effective volume decreases, which increases the activation of the angiotensin–aldosterone axis [20]. This stimulates renal sodium reabsorption, increasing plasma volume and preload to increase cardiac output. Owing to these effects of thyroid hormones on the heart and vessels, abnormal thyroid function could result in cardiovascular diseases, including angina, arrhythmia, and myocardial infarction [23]. The imbalance of oxygen supply and demand caused by the activation of the sympathetic nervous system and direct effect on its automaticity by abnormal thyroid function could explain them. The exact mechanism of coronary vasospasm owing to thyroid dysfunction has not yet been defined, but it is hypothesized that it might be associated with increased sensitivity to coronary artery vasoconstriction and decreased vasodilation. In addition, hyperthyroidism increases vasomotor activity, which has been demonstrated by increased endothelium-dependent flow-mediated vasodilation [9]. Interestingly, because thyroxine’s effect on peripheral circulation is vasodilation, it seems paradoxical that coronary artery vasospasm can be caused by hyperthyroidism although the coronary artery differs from peripheral vasculature.

It is noteworthy that an in vitro study has found that vasoconstrictive agents such as catecholamines and 5-hydroxytryptamine enhance the vascular smooth muscle contractions in thyrotoxic status [24]. In addition, hyperthyroidism is related to increased chronotropic sensitivity and upregulated numbers of adrenergic receptors, resulting in enhanced sympathoadrenal activity [8]. The effects of this hyperthyroid status may lead to vasospasm by sympathetic α-adrenergic receptor stimulation on coronary arteries [25].

### 4.1. Sex Differences in the Effects of Hyperthyroidism on Vasospasm

It is known that sex affects the prevalence of thyroid dysfunction [26], and the vascular benefits of estrogen are well known: inhibition of endothelial dysfunction, inhibition of vascular smooth muscle contraction and promotion of endothelium-dependent relaxation by increasing prostacyclin and nitric oxide [27,28]. Indeed, the interactions between thyroid and sex hormones occur through the hypothalamic–pituitary–gonadal and the hypothalamic–pituitary–thyroid axes [29]. Thus, we speculated that hyperthyroidism may influence these beneficial vascular effects of estrogen by interfering with the hypothalamic–pituitary–gonadal axis. In our study, the effect of hyperthyroidism on VA was greater in women than in men, although there was no statistical significance. Lee et al. [30] showed that there were significantly more women in the coronary vasospasm with thyrotoxicosis group than the coronary vasospasm without thyrotoxicosis group (62.5% vs. 32.2%, *p* = 0.001).

### 4.2. Clinical Significance and Implications

Our study demonstrated for the first time that hyperthyroidism could be a potential risk for VA occurrence independent of other risk factors like male sex and smoking, especially in females.

As far as we know, there have been no previous studies regarding the relationship between hyperthyroidism and VA occurrence and prognosis. There have only been case reports, mostly including women, and only one single center retrospective study [30] including only 32 patients with thyrotoxicosis and VA, which argued that more severe presentation of spasm was found in the thyrotoxicosis group. That study did not investigate the association of VA occurrence with the thyrotoxicosis but just showed the clinical presentation of thyrotoxicosis patients with VA. In this regard, our study has value in its first investigation on this subject and its large scale including 831 VA patients.

From a clinical viewpoint, our study results could help in real world practice. When we encounter a patient with hyperthyroidism complaining of chest pain, we should suspect VA, especially in women, even in the case that the nature of chest pain is ambiguous and far from atherosclerotic ischemic angina pain. From the prognosis viewpoint, we have demonstrated the similar clinical outcomes at 2 year follow up between hyperthyroidism and non-hyperthyroidism patients with VA. Thus, hyperthyroidism patients with VA could be educated and given information on the benign prognosis. Further study investigating whether treatment of hyperthyroidism in VA patients could relieve symptoms and affect the prognosis is needed.

### 4.3. Study Limitations

Several limitations of this study must be considered. First, this is a prospective multicenter cohort study; in contrast to a randomized controlled trial, this study may have inevitable bias that could affect the results. Second, for evaluating the independent association between hyperthyroidism and VA development, there may be hidden confounding factors. In multivariable logistic regression analysis, we adjusted possible individual confounders, but the difference in the OR of variables between univariate analysis and multivariable analysis was over 10%. Third, while evaluating the association between VA and hyperthyroidism, especially in women, there was a lack of data on obstetric history, menopausal status, as well as post-menopausal hormone replacement or oral contraceptives use that affect thyroid function. Fourth, the data on treatment of hyperthyroidism, serial follow-up data for thyroid function, and change in VA symptoms following treatment for hyperthyroidism were not presented, and timely treatment after the diagnosis of hyperthyroidism at the time of hospitalization could affect the cardiovascular events. These are challenging points that make it impossible to establish a cause–effect relationship between VA and hyperthyroidism. However, our findings may help clinicians to emphasize and remind them that they should suspect VA when they encounter a patient with hyperthyroidism complaining of chest pain in actual practice. Finally, there were fewer women and men with hyperthyroidism than those without hyperthyroidism. This may limit the interpretation of our results.

## 5. Conclusions

Hyperthyroidism was independently associated with the occurrence of VA and was pronounced in women. The prognosis of VA patients with hyperthyroidism was similar to VA patients without hyperthyroidism. Clinicians should consider thyroid function status in suspecting and diagnosing VA in patients complaining of chest pain.

## Figures and Tables

**Table 1:** Baseline Characteristics.

	All (*n* = 1239)	VA (*n* = 831)	Non-VA (*n* = 408)
Age, years	55.4 ± 11.7	55.3 ± 11.4	55.4 ± 12.4
Male, *n* (%)	629 (50.8)	485 (58.4)	144 (35.3)
BMI, kg/m^2^^†^	24.4 (22.5–26.7)	24.4 (22.5–26.6)	24.5 (22.6–26.8)
Previous CAD, *n* (%)	133 (10.7)	97 (11.7)	36 (8.8)
Diabetes mellitus, *n* (%)	115 (9.3)	69 (8.3)	46 (11.3)
Hypertension, *n* (%)	463 (37.4)	317 (38.1)	146 (35.8)
Dyslipidemia, *n* (%)	168 (13.6)	105 (12.7)	63 (15.4)
Alcohol drinking, *n* (%)	449 (36.2)	326 (39.2)	123 (30.1)
Current smoking, *n* (%)	272 (22.0)	224 (27.1)	48 (11.8)
**Laboratory finding**			
TSH, μIU/mL ^†^	1.6 (1.0–2.4)	1.5 (1.0–2.3)	1.7 (1.1–2.6)
Free T4, ng/dL ^†^	1.2 (1.0–1.3)	1.2 (1.1–1.4)	1.2 (1.0–1.3)
T3, ng/dL ^†,‡^	2.1 (1.4–99.7)	2.0 (1.4–99.4)	2.3 (1.5–100.4)
Hyperthyroidism, *n* (%)	98 (7.9)	83 (10.0)	15 (3.7)
Hypothyroidism, *n* (%)	59 (4.8)	42 (5.1)	17 (4.2)
LVEF, % ^†^	64.0 (61.2–67.9)	64.0 (61.0–68.0)	64.2 (61.5–67.7)
**Previous cardiovascular medication**			
Antiplatelet, *n* (%)	225 (18.2)	163 (19.6)	62 (15.2)
Stains, *n* (%)	166 (13.4)	105 (12.6)	61 (15.0)
CCBs, *n* (%)	219 (17.7)	153 (18.4)	66 (16.2)
**Clinical diagnosis before ergonovine provocation test**			
Angina, *n* (%)	1087 (87.7)	722 (86.9)	365 (89.5)
Myocardial infarction, *n* (%)	22 (1.8)	18 (2.2)	4 (1.0)
Cardiac arrest, *n* (%)	8 (0.6)	6 (0.7)	2 (0.5)
Syncope, *n* (%)	18 (1.5)	10 (1.2)	8 (2.0)
VT or VF, *n* (%)	6 (0.5)	4 (0.5)	2 (0.5)
AV block, *n* (%)	1 (0.1)	1 (0.1)	0 (0.0)

^†^ Continuous variables with non-normal distribution presented as median (interquartile range). ‡ The data of T3 was available in 820 patients. AV, atrio-ventricular; BMI, body mass index; CAD, coronary artery disease; CCB, calcium-channel blocker; LVEF, left ventricular ejection fraction; T4, thyroxine 4; TSH, thyroid-stimulating hormone; VA, vasospastic angina; VF, ventricular fibrillation; VT, ventricular tachycardia.

**Table 2:** Predictors of Vasospastic Angina.

	Univariate			Multivariable		
	OR	95% CI	*p*	OR	95% CI	*p*
Hyperthyroidism *	2.91	1.655–5.106	<0.001	3.27	1.811–5.897	<0.001
TSH, μIU/mL	0.98	0.946–1.008	0.137	-	-	-
Free T4, ng/dL	1.22	1.092–1.362	<0.001	-	-	-
T3, ng/dL	1.00	0.997–1.003	0.953	-	-	-
Sex, male	2.57	2.011–3.285	<0.001	2.19	1.644–2.907	<0.001
Age	1.00	0.989–1.009	0.892	-	-	-
Previous CAD	1.37	0.913–2.042	0.129	-	-	-
Hypertension	1.11	0.865–1.416	0.419	-	-	-
Diabetes	0.71	0.481–1.056	0.091	-	-	-
Dyslipidemia	0.80	0.567–1.115	0.184	-	-	-
Current smoking	2.78	1.981–3.896	<0.001	1.92	1.311–2.804	0.001
Alcohol drinking	1.50	1.161–1.927	0.002	0.91	0.675–1.218	0.515

* The multivariate logistic regression analysis was performed with variables with predictive significance (*p* < 0.05) of VA in univariate analysis except free T4 concentration. CAD, coronary artery disease; CI, confidence interval; OR, odds ratio; T3, triiodothyronine; T4, thyroxine; TSH, thyroid stimulating hormone.

**Table 3:** Clinical Event Rate of Patients with Vasospastic Angina According to the Presence or Absence of Hyperthyroidism.

	VA Group			
	All (*n* = 831)	Presence of Hyperthyroidism (*n* = 83)	Absence of Hyperthyroidism (*n* = 748)	*p* Value
Composite events	30 (3.6)	2 (2.4)	28 (3.7)	0.760
Cardiac death	2 (0.2)	0 (0.0)	2 (0.3)	>0.999
ACS	24 (2.9)	2 (2.4)	22 (2.9)	>0.999
VT or VF	2 (0.2)	0 (0.0)	2 (0.3)	>0.999
AV block	3 (0.4)	0 (0.0)	3 (0.4)	>0.999
All-cause death	4 (0.5)	0 (0.0)	4 (0.5)	>0.999

ACS, acute coronary syndrome; AV, atrioventricular; VF, ventricular fibrillation; VA, vasospastic angina; VT, ventricular tachycardia.

## Supplementary Materials

The following are available online at https://www.mdpi.com/2077-0383/9/9/3020/s1, Table S1: Baseline characteristics of patients with and without vasospastic angina according to sex, Table S2: Predictor of vasospastic angina in men and women, Table S3: Clinical event rate of patients with vasospastic angina according to sex, Figure S1: The cumulative composite clinical event-free survival rates according hyperthyroidism status
