# Supplementary material for: Hyperthyroidism Is Associated with the Development of Vasospastic Angina, but Not with Cardiovascular Outcomes
Source: J Clin Med. 2020 Sep 19;9(9):3020. doi: 10.3390/jcm9093020 (PMC7565955; doi:10.3390/jcm9093020)
Supplement: Supplementary file 1 [file jcm-09-03020-s001.pdf]

Supplementary Materials

Hyperthyroidism is Associated with Development of Vasospastic Angina, but Not with Clinical Outcomes

Hyun-Jin Kim, MD[1]; Sang-Ho Jo, MD[2*]; Min-Ho Lee, MD[3]; Won-Woo Seo, MD[4]; Sang Hong Baek[5]

[1]Division of Cardiology, Department of Internal Medicine, Hanyang University College of Medicine, Seoul, Korea, [2]Cardiovascular center, Hallym University Sacred Heart Hospital, Anyang-si, Korea, [3] Division of Cardiology, Department of Internal Medicine, Soonchunhyang University Seoul Hospital, Seoul, Korea, [4]Division of Cardiology, Department of Internal Medicine, Kangdong Sacred Heart Hospital, Hallym University College of Medicine, Seoul, Korea, [5]Division of Cardiology, Seoul St. Mary's Hospital, The Catholic University of Korea, Seoul, South Korea

Address for Correspondence

Sang-Ho Jo, MD, PhD

Division of Cardiology, Department of Internal Medicine, Hallym University College of Medicine

Hallym University Sacred Heart Hospital

22, Gwanpyeong-ro 170beon-gil, Dongan-gu, Anyang-si, Gyeonggi-do, Republic of Korea

Tel: +82-031-380-3722

E-mail: sophi5neo@gmail.com

Table S1. Baseline characteristics of patients with and without vasospastic angina according to sex.

	Men				*Women*			
	All (n = *629)*	*VA* (n = *485)*	*Non-VA* (n = *144)*	*p value*	*All* (n = *610)*	*VA* (n = *346)*	*Non-VA* (n = *264)*	*p value*
Age, years	53.6 ± 12.3	54.5 ± 11.8	50.4 ± 13.3	<0.001	57.2 ± 10.8	56.5 ± 10.6	58.1 ± 11.0	0.068
BMI, kg/m^{2†}	24.5 (22.7-26.5)	24.4 (22.5-26.5)	24.6 (23.2-26.7)	0.215	24.4 (22.2-26.8)	24.4 (22.3-26.8)	24.3 (22.2-26.9)	0.801
Previous CAD, n (%)	68 (10.8)	62 (12.8)	6 (4.2)	0.004	65 (10.7)	35 (10.1)	30 (11.4)	0.621
Diabetes mellitus, n (%)	55 (8.8)	43 (8.9)	12 (8.4)	0.860	60 (9.8)	26 (7.5)	34 (12.9)	0.028
Hypertension, n (%)	227 (36.1)	188 (38.8)	39 (27.3)	0.012	235 (38.5)	129 (37.3)	106 (40.2)	0.471
Dyslipidemia, n (%)	68 (10.8)	50 (10.3)	18 (12.6)	0.645	100 (16.4)	55 (15.9)	45 (17.0)	0.438
Alcohol drinking, n (%)	351 (55.9)	271 (55.9)	80 (55.9)	0.989	98 (16.1)	55 (15.9)	43 (16.3)	0.896
Current smoking, n (%)	249 (39.9)	205 (42.6)	44 (30.8)	0.011	23 (3.8)	19 (5.5)	4 (1.5)	0.010
Laboratory finding								
TSH, μIU/mL†	1.4 (0.9-2.2)	1.4 (0.9-2.1)	1.5 (1.0-2.3)	0.245	1.8 (1.1-2.9)	1.8 (1.1-2.7)	1.8 (1.1-2.9)	0.437
Free T4, ng/dL†	1.2 (1.1-1.4)	1.2 (1.1-1.4)	1.2 (1.1-1.3)	0.375	1.2 (1.0-1.3)	1.2 (1.0-1.4)	1.1 (1.0-1.3)	0.006
T3, ng/dL†	75.4 (1.4-106.6)	76.0 (1.4-103.5)	67.5 (1.5-104.3)	0.426	1.9 (1.4-94.1)	1.9 (1.4-89.1)	2.0 (1.4-97.1)	0.484
Hyperthyroidism, n (%)	48 (7.6%)	42 (8.7%)	6 (4.2%)	0.077	50 (8.2%)	41 (11.8%)	9 (3.4%)	<0.001
Hypothyroidism, n (%)	18 (2.9%)	14 (2.9%)	4 (2.8%)	0.999	41 (6.7%)	28 (8.1%)	13 (4.9%)	0.143
LVEF, %†	63.7 (60.8-67.1)	63.6 (61.0-67.1)	63.9 (60.7-67.0)	0.717	64.7 (61.6-68.0)	64.4 (61.3-68.1)	65.0 (62.0-68.0)	0.519
Previous cardiovascular medication								
Antiplatelet, n (%)	125 (19.9)	109 (22.5)	16 (11.2)	0.008	99 (16.2)	54 (15.6)	45 (17.0)	0.521
Stains, n (%)	76 (12.1)	60 (12.4)	16 (11.2)	0.235	90 (14.8)	45 (13.0)	45 (17.0)	0.124
CCBs, n (%)	110 (17.5)	95 (19.6)	15 (10.5)	0.021	109 (17.9)	58 (16.8)	51 (19.3)	0.425
Clinical diagnosis before ergonovine provocation test								

Angina, n (%)	540 (86.0)	416 (85.8)	124 (86.7)	0.840	546 (89.6)	240 (90.9)	306 (88.4)	0.324
Myocardial infarction, n (%)	15 (2.4)	13 (2.7)	3 (1.4)	0.539	7 (1.1)	5 (1.4)	2 (0.8)	0.705
Cardiac arrest, n (%)	7 (1.1)	5 (1.0)	2 (1.4)	0.807	1 (0.2)	1 (0.3)	0 (0.0)	>0.999
Syncope, n (%)	12 (1.9)	7 (1.4)	5 (3.5)	0.250	6 (1.0)	3 (0.9)	3 (1.1)	>0.999
VT or VF, n (%)	6 (1.0)	4 (0.8)	2 (1.4)	0.713	0 (0.0)	0 (0.0)	0 (0.0)	-
AV block, n (%)	1 (0.2)	1 (0.2)	0 (0.0)	0.744	0 (0.0)	0 (0.0)	0 (0.0)	

AV, atrio-ventricular; BMI, body mass index; CAD, coronary artery disease; CCB, calcium-channel blocker; LV EF, left ventricular ejection fraction; T4, thyroxine 4; TSH, thyroid stimulating hormone; VA, vasospastic angina; VF, ventricular fibrillation; VT, ventricular tachycardia. † Continuous variables with non-normal distribution presented as median (interquartile range).

.

Table S2. Predictor of vasospastic angina in men and women.

	Men						Women					
	Univariate			Multivariate			Univariate			Multivariate		
	OR	95% CI	*P*	OR	95% CI	*P*	OR	95% CI	*P*	OR	95% CI	*P*
Hyperthyroidism	2.17	0.901-5.201	0.084	-	-	-	3.81	1.816-7.986	<0.001	4.38	2.011-9.537	<0.001
Age	1.03	1.011-1.043	0.001	1.03	1.010-1.044	0.002	1.01	0.999-1.029	0.069	1.01	0.991-1.023	0.379
Previous CAD	3.37	1.427-7.964	0.006	3.28	1.372-7.860	0.008	0.88	0.524-1.471	0.621	-	-	-
Hypertension	1.65	1.095-2.475	0.017	1.36	0.880-2.103	0.167	0.89	0.638-1.231	0.471	-	-	-
Diabetes	1.07	0.548-2.089	0.843	-	-	-	0.55	0.321-0.941	0.029	0.57	0.327-0.998	0.049
Dyslipidemia	0.81	0.454-1.432	0.463	-	-	-	0.93	0.602-1.426	0.727	-	-	-
Current smoking	1.69	1.134-2.513	0.010	2.14	1.410-3.242	<0.001	3.76	1.264-11.195	0.017	4.04	1.336-12.205	0.013
Alcohol drinking	1.01	0.697-1.473	0.946	-	-	-	0.97	0.628-1.502	0.896	-	-	-

CAD, coronary artery disease; CI, confidence interval; OR, odds ratio.

Table S3. Clinical event rate of patients with vasospastic angina according to sex

	Men with VA				Women with VA			
	All (n = 485)	Presence of hyperthyroidism (n = 42)	Absence of hyperthyroidism (n = 443)	p value	All (n = 346)	Presence of hyperthyroidism (n = 41)	Absence of hyperthyroidism (n = 305)	p value
Composite events	17 (3.5)	1 (2.4)	16 (3.6)	>0.999	13 (3.8)	1 (2.4)	12 (3.9)	>0.999
Cardiac death	2 (0.4)	0 (0.0)	2 (0.5)	>0.999	0 (0.0)	0 (0.0)	0 (0.0)	-
ACS	12 (2.5)	1 (2.4)	11 (2.5)	>0.999	12 (3.5)	1 (2.4)	11 (3.6)	>0.999
VT or VF	2 (0.4)	0 (0.0)	2 (0.5)	>0.999	0 (0.0)	0 (0.0)	0 (0.0)	-
AV block	1 (0.2)	0 (0.0)	1 (0.2)	>0.999	2 (0.6)	0 (0.0)	2 (0.7)	>0.999
All-cause death	3 (0.6)	0 (0.0)	3 (0.7)	>0.999	1 (0.3)	0 (0.0)	1 (0.3)	>0.999

ACS, acute coronary syndrome; AV, atrioventricular; VF, ventricular fibrillation; VT, ventricular tachycardia

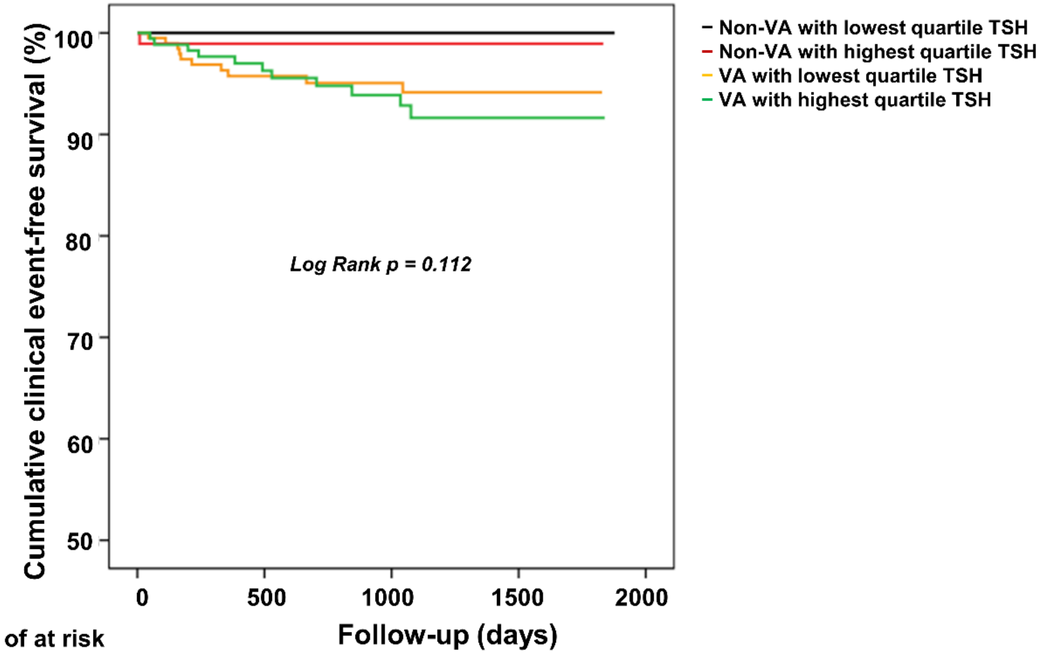

Numbers of at risk

Non-VA with lowest quartile TSH	97	41	29	6	0
Non-VA with highest quartile TSH	118	61	43	14	0
VA with lowest quartile TSH	240	143	106	16	0
VA with highest quartile TSH	198	133	95	20	0

(A) Highest and lowest TSH quartile in both VA and non-VA group

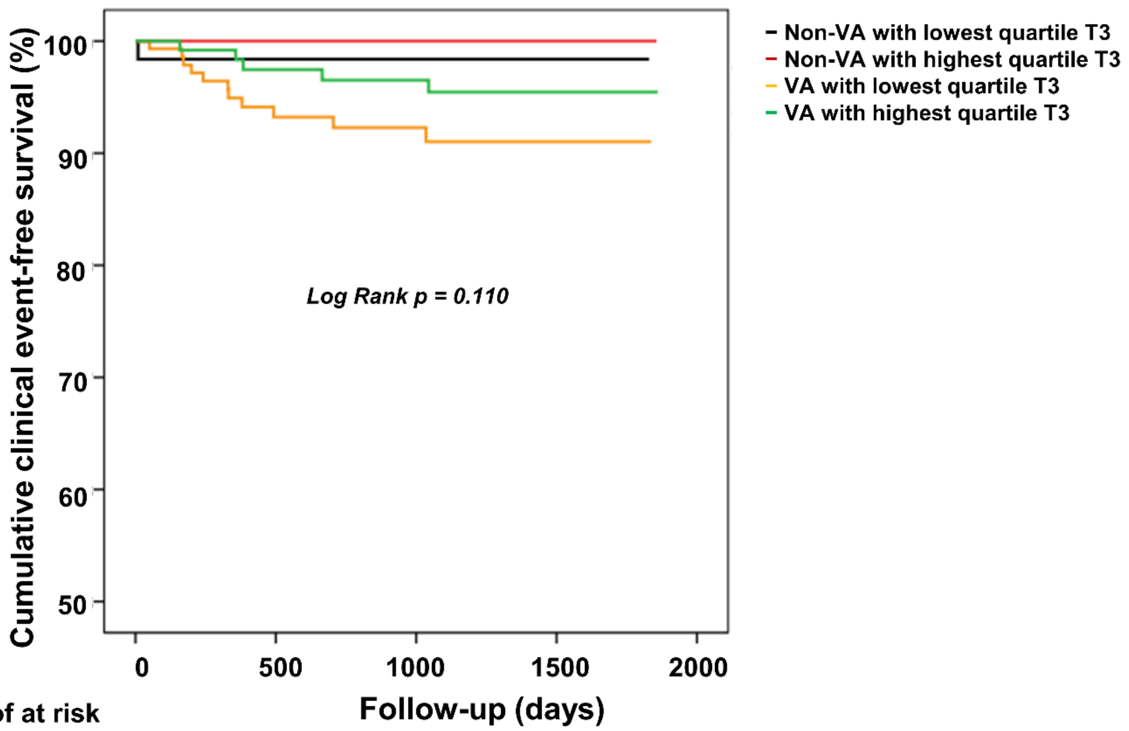

Numbers of at risk

Non-VA with lowest quartile T3	64	37	18	2	0
Non-VA with highest quartile T3	67	33	32	9	0
VA with lowest quartile T3	168	104	77	1	0
VA with highest quartile T3	138	105	94	25	0

(B) Highest and lowest T3 quartile in both VA and non-VA group

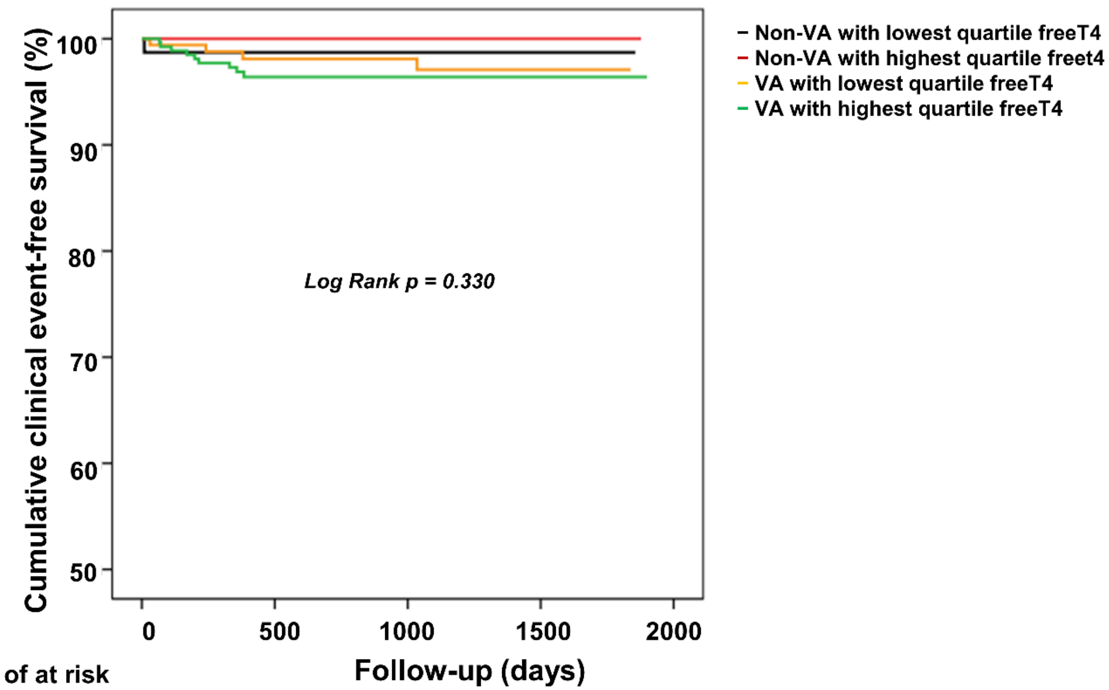

Numbers of at risk

Non-VA with lowest quartile freeT4	118	53	33	11	0
Non-VA with highest quartile freeT4	140	63	44	12	0
VA with lowest quartile freeT4	185	133	101	11	0
VA with highest quartile freeT4	329	182	139	28	0

(C) Highest and lowest free-T4 quartile in both VA and non-VA group

Figure S1. The cumulative composite clinical event-free survival rates according hyperthyroidism status
